# Supplementary material for: Flower lose, a cell fitness marker, predicts COVID‐19 prognosis
Source: EMBO Mol Med. 2021 Oct 18;13(11):e13714. doi: 10.15252/emmm.202013714 (PMC8573598; doi:10.15252/emmm.202013714)
Supplement: Supplementary file 1 — Appendix [file EMMM-13-e13714-s002.doc]

**Flower Lose, a Cell Fitness Marker, Predicts COVID-19 Prognosis**

Michail Yekelchyk^1,#^, Esha Madan^2,#^, Jochen Wilhelm^3,4,@^, Kirsty R Short^5,@^, António M. Palma^2,@^, Linbu Liao^6,@^, Denise Camacho^2^, Everlyne Nkadori^7^, Michael T. Winters^8^, Emily S. Westemeier^8^, Inês Rolim^2^, Raquel Cruz-Duarte^9^, Christopher J. Pelham^10^, Masaki Nagane^11^, Kartik Gupta^12^, Sahil Chaudhary^12^, Thomas Braun^1,13^, Raghavendra Pillappa^14^, Mark S. Parker^15^, Thomas Menter^16^, Matthias Matter^17^, Jasmin Dionne Haslbauer^17^, Markus Tolnay^18^, Kornelia D. Galior^7^, Kristina A. Matkwoskyj^7^, Stephanie M. McGregor^7^, Laura K. Muller^7^, Emad A Rakha^19^, Antonio Beltran^2^, Ronny Drapkin^20, 21, 22^, Maximilian Ackermann^23, 24^, Paul B. Fisher^25, 26, 27^, Steven R Grossman^28, 29^, Andrew K. Godwin^30, 31^, Arutha Kulasinghe^32^, Ivan Martinez^8^, Clay B Marsh^8^, Benjamin Tang^33^, Max S Wicha^34, 35^, Kyoung Jae Won^6, 36^, Alexandar Tzankov^17,$^, Eduardo Moreno^2,$^, Rajan Gogna^6, $^

**^1^**Department of Cardiac Development and Remodelling, Max Planck Institute for Heart and Lung Research, 61231, Bad Nauheim, Germany.

**^2^**Champalimaud Centre for the Unknown, 1400-038 Lisbon, Portugal.

**^3^**Justus-Liebig-University, Universities Giessen & Marburg Lung Center, German Center for Lung Research (DZL), Germany;

**^4^**Institute for Lung Health (ILH), Justus-Liebig-University Giessen, Universities Giessen & Marburg Lung Center, German Center for Lung Research (DZL), Germany.

**^5^**School of Chemistry and Molecular Biosciences, The University of Queensland, Brisbane, Australia.

**^6^**Biotech Research and Innovation Centre (BRIC), University of Copenhagen, Ole Maaløes Vej 5, 2200 Copenhagen N, Denmark.

**^7^**Department of Pathology and Laboratory Medicine, University of Wisconsin Carbone Cancer Center, University of Wisconsin-Madison School of Medicine and Public Health, Madison, Wisconsin, USA.

**^8^**Department of Microbiology, Immunology & Cell Biology and WVU Cancer Institute, West Virginia University, Morgantown, WV 26506, USA.

**^9^**Instituto de Medicina Molecular João Lobo Antunes, Faculdade de Medicina, Universidade de Lisboa, Lisboa, 1649-028, Portugal.

**^10^**Eurofins Panlabs Inc., St. Charles, MO, USA.

**^11^**Department of Biochemistry, School of Veterinary Medicine, Azabu University, Kanagawa, Japan.

**^12^**Department of Surgery, School of Medicine and Public Health, University of Wisconsin, Madison, WI, USA.

**^13^**Member of the German Center for Cardiovascular Research (DZHK).

**^14^**Department of Pathology, Virginia Commonwealth University School of Medicine, Richmond, VA, 23298, USA.

**^15^**Department of Diagnostic Radiology and Internal Medicine, Early Detection Lung Cancer Screening Program, Thoracic Imaging Division, Thoracic Imaging Fellowship Program, VCU Health Systems, Richmond, VA 23298-0615, USA.

**^16^**Institute of Medical Genetics and Pathology, University Hospital Basel, University of Basel, Schönbeinstrasse 40, 4031, PathologyBasel, Switzerland.

**^17^**Institute of Pathology, University of Basel, Schönbeinstrasse 40, 4003 Basel, Switzerland.

**^18^**Institute for Medical Genetics and Pathology, Division of Neuropathology, University Hospital Basel, Basel, Switzerland.

**^19^**Department of Pathology, Division of Cancer and Stem Cells, School of Medicine, University of Nottingham, Nottingham University Hospitals, Nottingham, NG5 1PB, UK.

**^20^**Penn Ovarian Cancer Research Center, Department of Obstetrics and Gynecology, University of Pennsylvania Perelman School of Medicine, Philadelphia, PA 19104, USA.

**^21^**Graduate Program in Cell and Molecular Biology, University of Pennsylvania Perelman School of Medicine, Philadelphia, PA 19104, USA.

**^22^**Basser Center for BRCA, Abramson Cancer Center, University of Pennsylvania School of Medicine, Philadelphia, PA 19104, USA.

**^23^**Institute of Pathology and Molecular Pathology, Helios University Clinic Wuppertal, University of Witten/Herdecke, Wuppertal, Germany

**^24^**Institute of Functional and Clinical Anatomy, University Medical Center of the Johannes Gutenberg-University Mainz, Mainz, Germany

**^25^**Department of Human and Molecular Genetics, Virginia Commonwealth University, School of Medicine, Richmond, VA, USA.

**^26^**Massey Cancer Center, Virginia Commonwealth University, Richmond, VA, 23298, USA.

**^27^**Institute of Molecular Medicine, Department of Human and Molecular Genetics, Virginia Commonwealth University, School of Medicine, Richmond, VA, 23298, USA.

**^28^**Department of Internal Medicine, Keck School of Medicine, Norris Comprehensive Cancer Center,

**^29^**University of Southern California, 1450 Biggy Street, Health Sciences Campus, Los Angeles CA 90033, USA.

**^30^**Department of Pathology and Laboratory Medicine; University of Kansas Medical Center; Kansas City, KS USA;

**^31^**University of Kansas Cancer Center; Kansas City, KS, USA

**^32^**The University of Queensland Diamantina Institute, The University of Queensland, Brisbane, QLD, Australia.

**^33^**Department of Intensive Care Medicine, Nepean Hospital, Penrith, NSW, Australia.

**^34^**University of Michigan, Rogel Cancer Center, Ann Arbor, MI 48109, USA.

**^35^**Department of Internal Medicine, Michigan Medicine, University of Michigan, 1500 East Medical Center Drive, RCC 7314, Ann Arbor, MI, 48105, USA.

**^36^**Novo Nordisk Foundation Center for Stem Cell Biology, DanStem, Faculty of Health and Medical Sciences, University of Copenhagen, Blegdams vej 3B, 2200 Copenhagen N, Denmark.

**# -** These authors contributed equally to the first author position;

**@ -** These authors contributed equally to the second author position.

**$ -** Correspondence to.

**Dr. Rajan Gogna**

- Biotech Research and Innovation Centre (BRIC), University of Copenhagen, Ole Maaløes Vej 5, 2200 Copenhagen N, Denmark.

**Email:**

rajangogna@gmail.com, rajan.gogna@bric.ku.dk;

**Phone:**

+45 35331419 and +351 910225386.

**Dr. Alexandar Tzankov**

- Institute of Medical Genetics and Pathology, University Hospital Basel, 4031 Basel, Switzerland;

**Email:**

Alexandar.Tzankov@usb.ch;

**Phone:**

+41 612653229.

**Dr. Eduardo Moreno**

- Champalimaud Centre for the Unknown, 1400-038 Lisbon, Portugal;

**Email:**

eduardo.moreno@research.fchampalimaud.org;

**Phone:**

+351 210489380.

**Keywords:** COVID-19, cell-fitness, Flower, biomarker, prognosis.

**Table of content:**

Appendix Figure S1: *hFwe-Lose* expression in lung tissue increases with age and host comorbidities.

Appendix Figure S2: *hFwe-Lose* expression in lung tissue increases with age and host comorbidities.

Appendix Figure S3. Poor lung tissue health reflected by *hFwe-Lose* exacerbates viral-mediated apoptosis.

Appendix Table S1: The table shows sex, age, and comorbidities of patients, who died from COVID-19.

Appendix Table S2: The table summarizes statistical information about selected *hFwe-Lose* threshold levels in relation to COVID-19 outcome prediction.


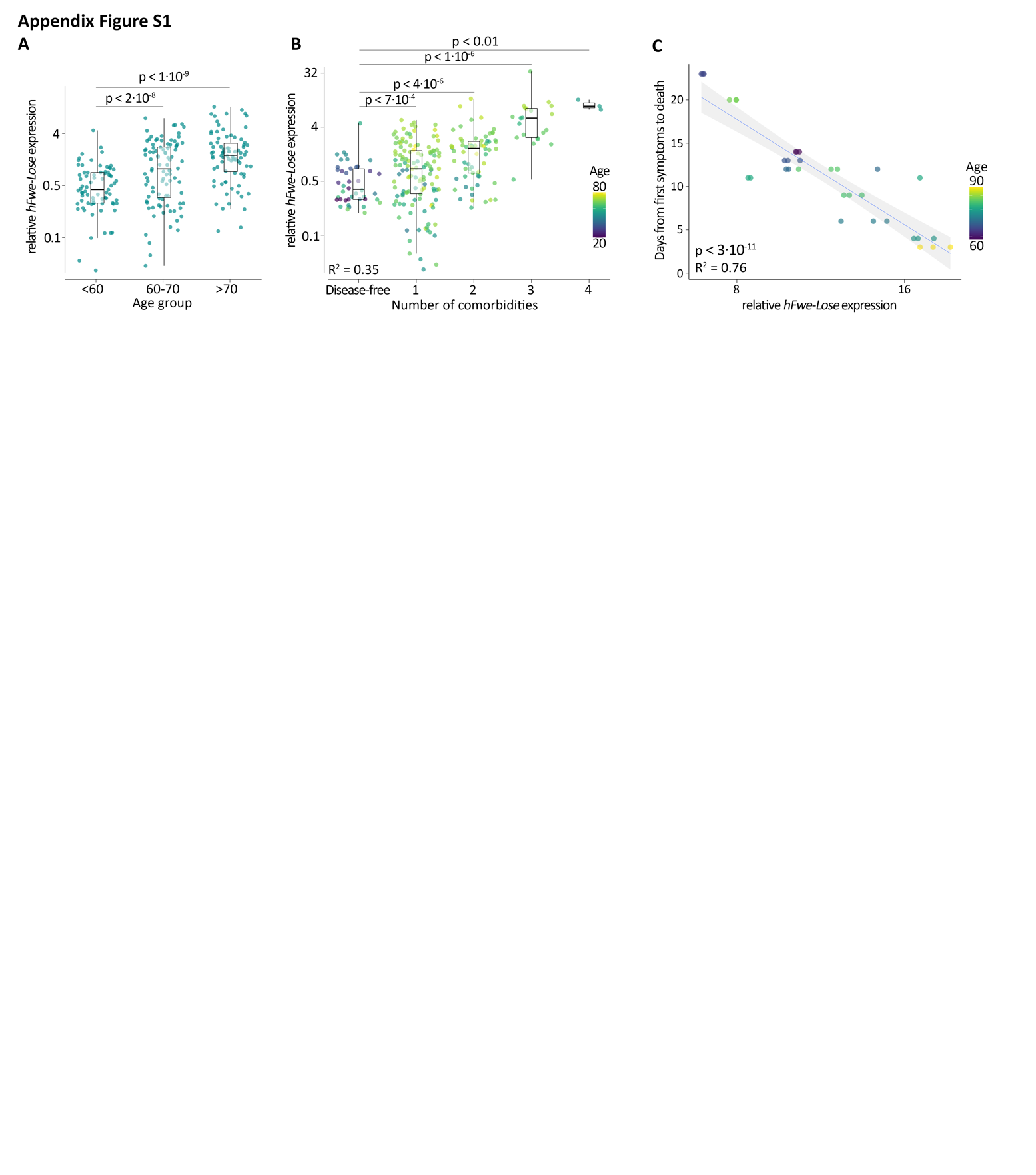


**Appendix Figure S1. *hFwe-Lose* expression in lung tissue increases with age and host comorbidities.**

**A |** *hFwe-Lose* biomarker expression is more abundant in elderly people. Patients over 70, as well as between 60- and 70-years old exhibit a significant upregulation of *hFwe-Lose* expression, compared to patients younger than 60 years. Two-sided Student’s t-test was performed, and p-values are presented on the plot. The vertical axis represents log2 expression quantification of *hFwe-Lose* according to the comparative Ct method. **B |** *hFwe-Lose* expression increases with total numbers of patients’ comorbidities. Box plot shows an increase of *hFwe- Lose* expression in the lung tissue of patients, suffering from multiple comorbidities. Pairwise two-sided Student’s t-test was performed, and p-values are presented on the plot. The vertical axis represents log2 expression quantification of *hFwe-Lose* according to the comparative Ct method. **C |** *hFwe-Lose* expression in autopsy lung tissue of patients who died from COVID-19 (n = 11). *hFwe-Lose* expression inversely correlates with the duration of the COVID-19 sickness (R2=0.76; slope CI - [24.9-32.1]). The vertical axis represents log2 expression quantification of *hFwe-Lose* expression. The horizontal axis represents the number of days from detection of first COVID-19 symptoms to the patient's death. Patient's age is depicted in color.

**
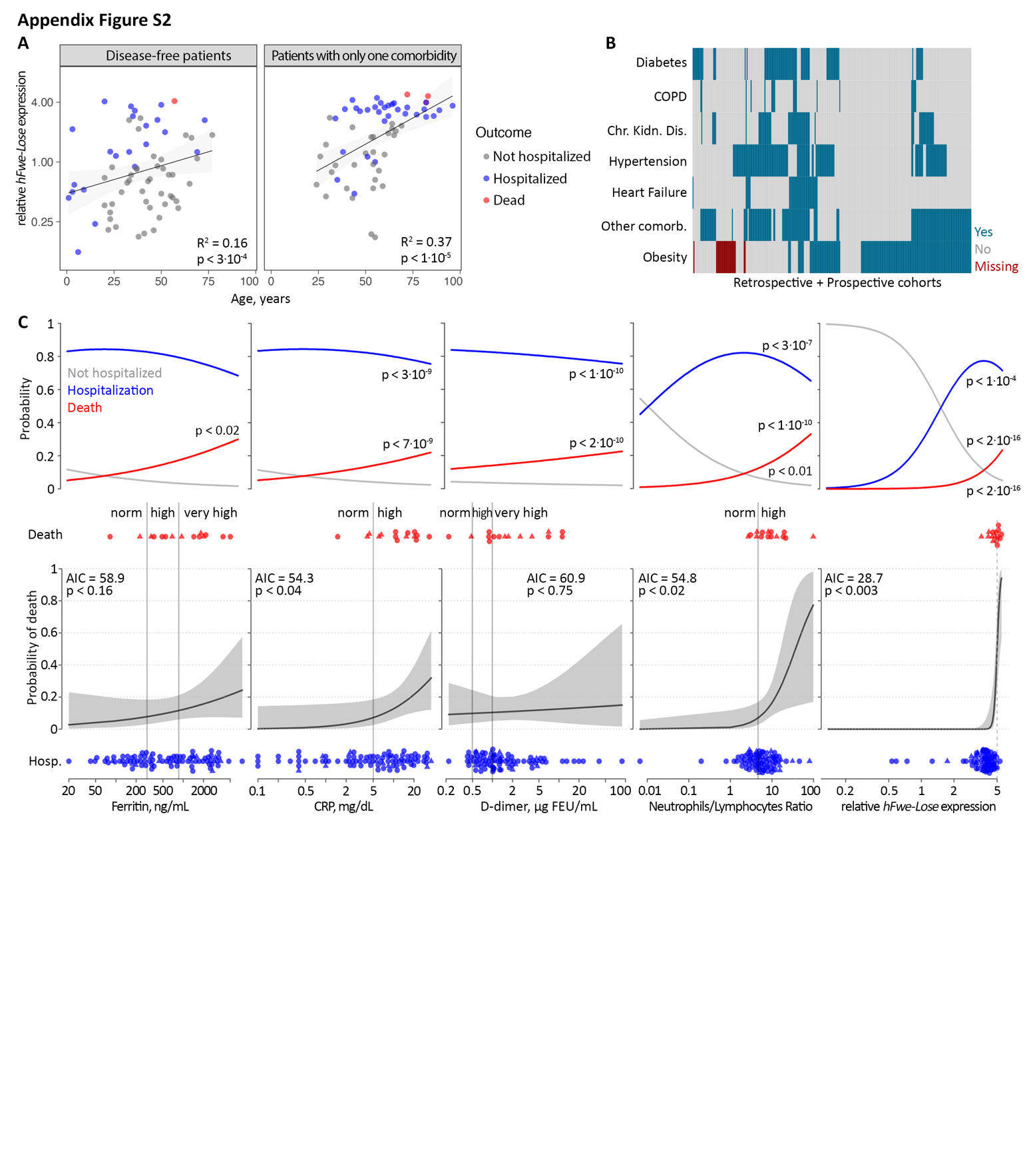
**

**Appendix Figure S2. *hFwe-Lose* expression in lung tissue increases with age and host comorbidities.**

**A |** *hFwe-Lose* biomarker expression is more abundant in nasal swab probes from elderly people in both disease-free individuals and patients with only one (any) comorbidity. *hFwe-Lose* expression was analyzed by RT-qPCR in 67 nasal swab samples taken from disease-free individuals and 66 patients with only one (any) comorbidity) with age between 1 and 96 years, taken at the very beginning of the disease (the earliest contact with physician, before the disease progression). The vertical axis represents log2 expression quantification of *hFwe-Lose* according to the comparative Ct method (normalized to non-hospitalized patients). Older patients show a significant upregulation of *hFwe-Lose* expression. Linear regression models prove a positive correlation between age and *hFwe-Lose* expression for three outcome groups: non-hospitalized (gray), hospitalized (blue) and deceased (red). R2= 0.16 (disease-free) and R2= 0.37 (one comorbidity); p (all curves) < 0.001. **B |** Many patients had more than one comorbidity at the same time. The heatmap shows the distribution of selected comorbidities (Diabetes, COPD, chronic kidney disease, hypertension, heart failure and obesity) across all patients (retrospective + prospective cohorts; n = 283; disease-free = 96, one comorbidity = 76, two comorbidities = 55, three comorbidities = 33, four comorbidities = 18, five comorbidities = 5). “Other comorbidity” refers to diseases or conditions, not directly associated with COVID-19 (cancer, Down syndrome, solid organ transplant, sickle cell disease, bone marrow transplant). Dark blue color refers to the presence of certain comorbidity, blue gray refers to its absence. Dark red color depicts missing patient’s data. **C |**The logistic models predict probability of any outcome based on known COVID-19 blood biomarkers (Ferritin, CRP, D- dimer and Neutrophils/Lymphocytes ratio) and *hFwe-Lose* expression in nasal swab samples (upper panel; p-values are presented on the plot). The logistic models predict probability of death based on known COVID-19 blood biomarkers (Ferritin, CRP, D-dimer and Neutrophils/Lymphocytes ratio) and *hFwe-Lose* expression in nasal swab samples of hospitalised patients (lower panel). The models also incorporate information about patients’ age, sex and comorbidities. These models show poor prediction capacity of known COVID-19 biomarkers (AIC = 58.9, p < 0.16; AIC = 54.3, p < 0.04; AIC = 60.9, p < 0.75; AIC = 54.8, p < 0.02; respectively, left to right), compared to the *hFwe-Lose* biomarker in the nasal swab samples (AIC = 28.7, p < 0.003).

**
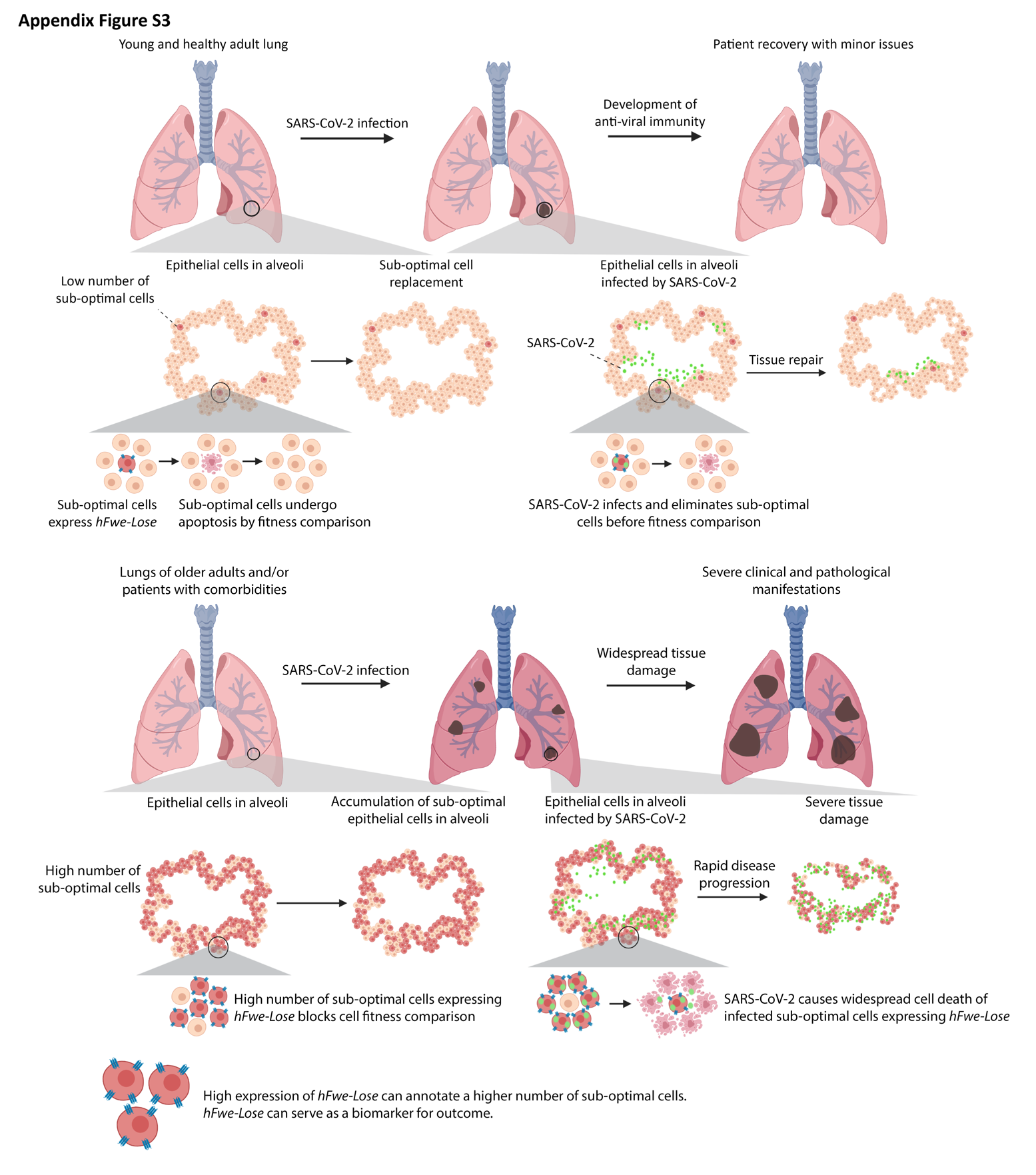
**

**Appendix Figure S3. Poor lung tissue health reflected by *hFwe-Lose* exacerbates viral-mediated apoptosis.**

Cellular fitness comparisons optimize tissue fitness and may alter the course of COVID-19 infection severity. Young and healthy adults are more likely to possess lung alveoli that contain a low percentage of suboptimal cells expressing *hFwe- Lose*. SARS-CoV-2 particles infecting highly competent lung tissue, the suboptimal cells are eliminated via apoptosis. The lower proportion of suboptimal cells attenuates alveolar damage, which slows disease progression and provides time to mount antiviral immune responses. The majority of young and middle-aged patients without comorbidities do not present severe clinical manifestations, and they recover with minor lung injuries.

Elderly adults, along with people who have comorbidities are more likely to possess lung alveoli that contain a higher percentage of suboptimal cells expressing *hFwe-Lose*. The greater proportion of suboptimal cells reduces the lung tissue’s ability to optimize tissue fitness. These tissues are more susceptible to infection by SARS-CoV-2, causing severe alveolar damage and swift disease progression. Severe clinical and pathological manifestations include pulmonary edema, pneumonia, and severe acute respiratory syndrome. Inhibition of apoptosis could improve survival of lung tissue. This information highlights the importance of the *hFwe-Lose* isoform as a biomarker and potential therapeutic target.

**
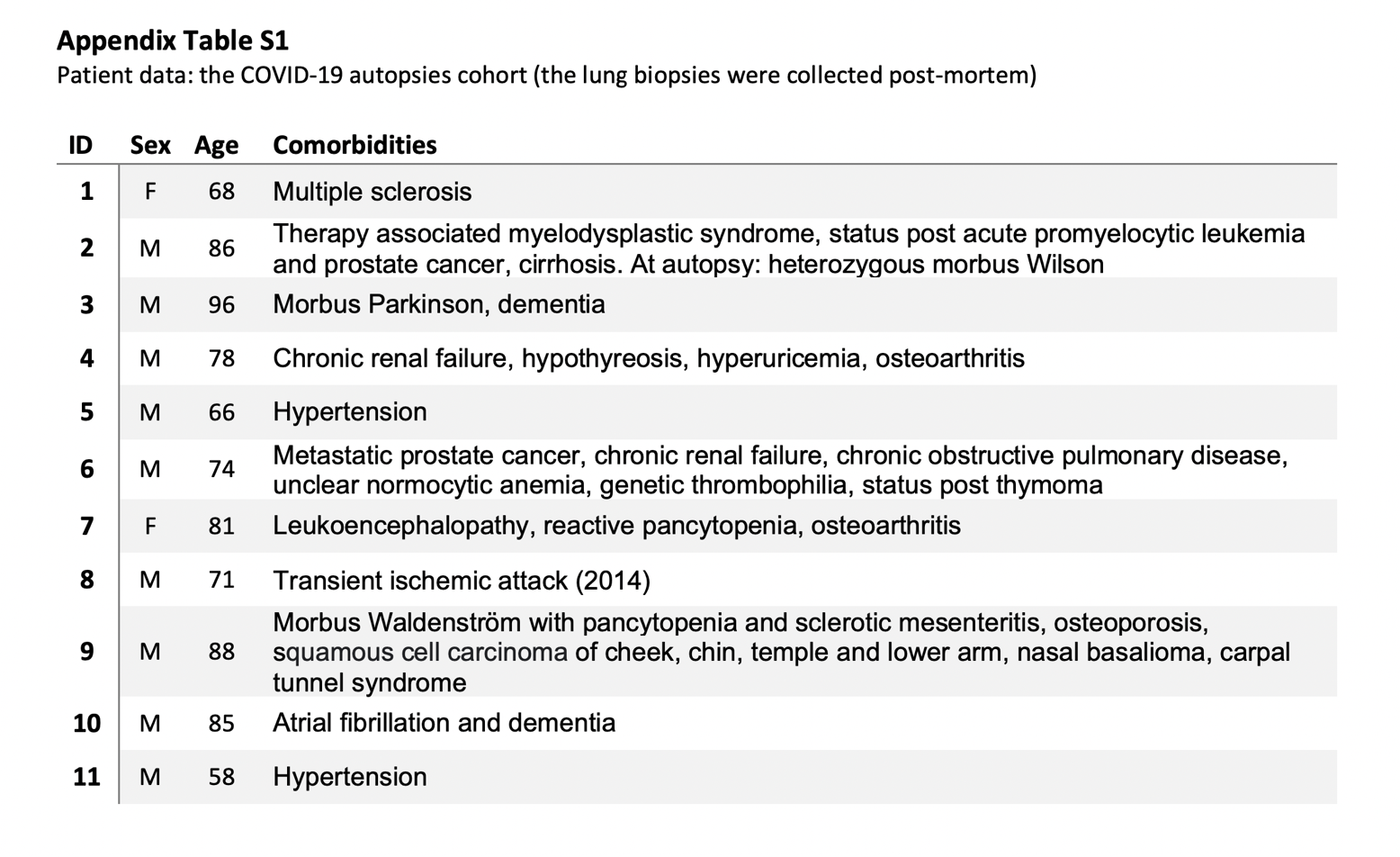
**

**Appendix Table S1.** The table shows sex, age and comorbidities of patients, who died from COVID-19. The lung biopsies were collected from these patients post-mortem to measure *hFwe-Lose* expression, as well as to evaluate histology of their lung tissue. The respective results are shown on Fig.1 and Fig. S1.

**
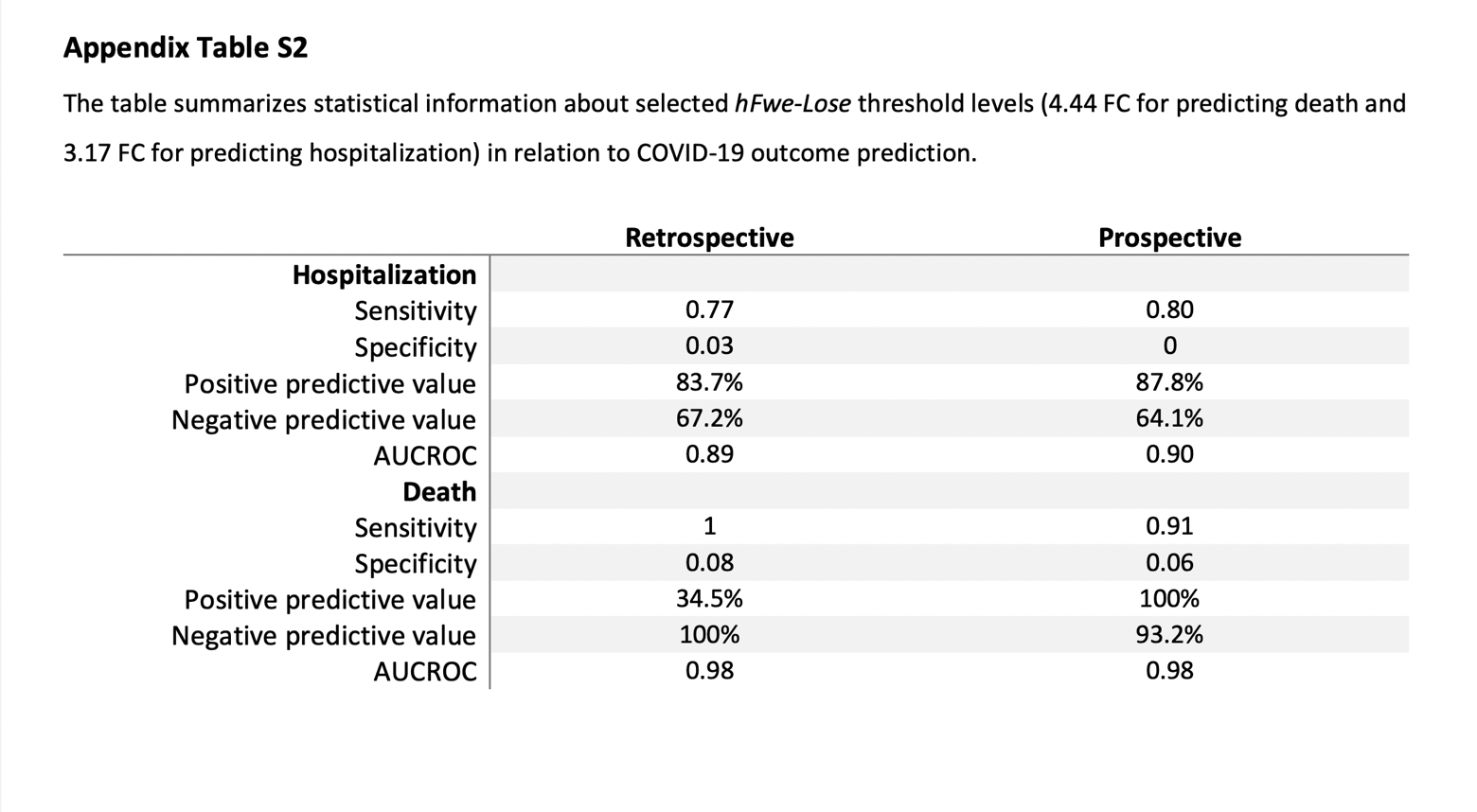
**

**Appendix Table S2.** The table summarizes statistical information about selected *hFwe-Lose* threshold levels (4.44 for predicting death and 3.17 for predicting hospitalization; both are FC, normalized to mean of non-hospitalized patients) in relation to COVID-19 outcome prediction. The table contains Sensitivity (TPR), Specificity (FPR), Positive and Negative Predictive values (PPV & NPV), and AUROC. The respective results are visualized on Fig.3F.
